# High Deuteration of Methanol in L1544

**DOI:** 10.1021/acsearthspacechem.5c00187

**Published:** 2025-12-22

**Authors:** Silvia Spezzano, Wiebke Riedel, Paola Caselli, Olli Sipilä, Yuxin Lin, Hayley A. Bunn, Elena Redaelli, Laurent H. Coudert, Andrés Megías, Izaskun Jimenez-Serra

**Affiliations:** † 28279 Max-Planck-Institut für Extraterrestrische Physik, Giessenbachstrasse 1, 85748 Garching, Germany; ‡ European Southern Observatory, Karl-Schwarzschild-Strasse 2, 85748 Garching, Germany; § Institut des Sciences Moléculaires d’Orsay (ISMO), CNRS, Université Paris-Saclay, F-91405 Orsay, France; ∥ Centro de Astrobiología (CAB), CSIC-INTA, Carretera de Ajalvir, km 4, 28805 Torrejón de Ardoz, Spain

**Keywords:** isotopic fractionation, deuteration maps, complex organic molecule, H-abstraction processes, non-LTE effects, L1544

## Abstract

Isotopic fractionation is a very powerful tool to follow the evolution of material from one stage to the next in the star-formation process. Prestellar cores exhibit some of the highest levels of deuteration because their physical conditions (*T* ≤ 10 K and *n*(H_2_) ≥ 10^5^ cm^–3^) greatly favor deuteration processes. Deuteration maps are a measure of the effectiveness of the deuteration across the core, and they are useful to study both the deuteration and the formation mechanism (either in the gas-phase or on grain surfaces) of the main species. Methanol is the simplest complex organic molecule (COM) that is O-bearing and detected in the interstellar medium (ISM). It represents the beginning of molecular complexity in star-forming regions; thus, a complete understanding of its formation and deuteration is a necessary step to understand the development of further chemical complexity. In this paper, we use single-dish observations with the IRAM 30 m telescope and state-of-the-art chemical models to investigate the deuteration of methanol toward the prototypical prestellar core L1544. We also compare the results of the chemical models with previous observations of deuterated methanol toward the presttellar cores HMM1 and L694-2. The spectra extracted from the CHD_2_OH map show that the emission is concentrated in the center and toward the northwest of the core. Using deep observations toward the dust and the methanol peaks of the core, we derive a very large deuterium fraction for methanol (∼20%) toward both peaks. The comparison of our observational results with chemical models has highlighted the importance of H-abstraction processes in the formation and deuteration of methanol. Deep observations combined with state-of-the-art chemical models are of fundamental importance in understanding the development of molecular complexity in the ISM. Our analysis also shows the importance of non-LTE effects when measuring the D/H ratios in methanol.

## Graphical abstract

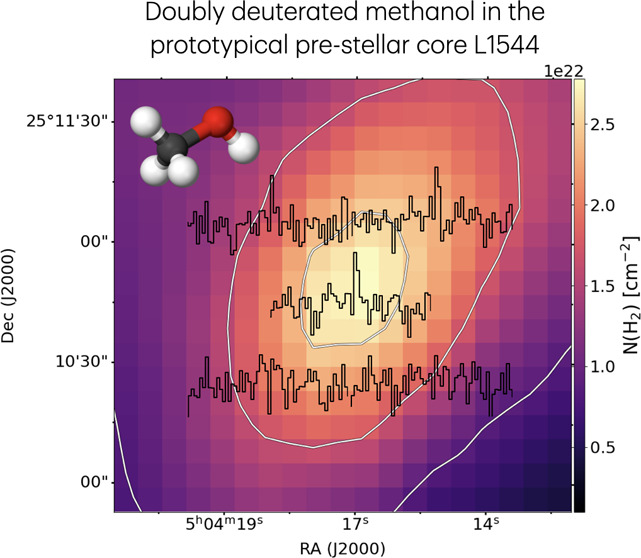

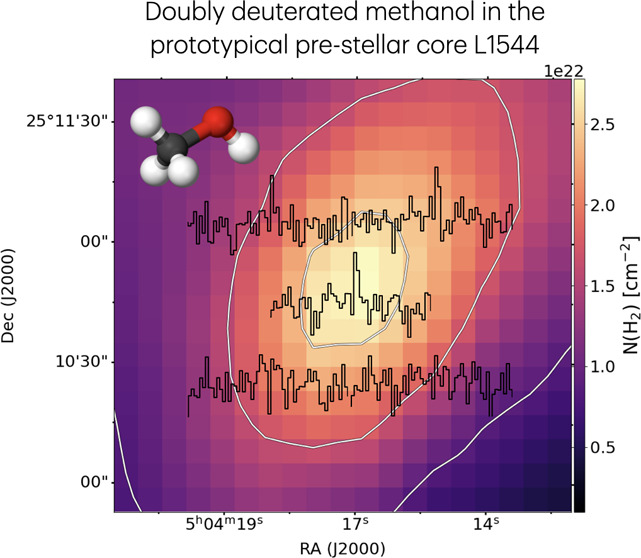

## 1 Introduction

Low-mass stars are formed by the collapse of dense cores within filamentary structures in molecular clouds.^1,2^ Dense cores are therefore crucial players in our understanding of the physical and chemical conditions at the dawn of star-formation. Prestellar cores are dynamically evolved starless cores with centrally concentrated density profiles and central densities higher than a few 10^5^ cm^–3^.^3,4^ Prestellar cores are of particular importance in the quest of understanding the initial conditions of low-mass star-formation because they are unstable against gravitational collapse and hence will certainly form a protostellar system. Conversely, starless cores that are less dense and not centrally concentrated (e.g., B68 and TMC-1 ^5,6^ ) might eventually evolve into a prestellar core and finally form a protostar or dissolve back into the interstellar medium.

The density structure of prestellar cores is generally modeled with a Bonnor-Ebert (BE) sphere^7,8^ with a central plateau and a density decrease outward that scales with *r* ^–2^ where the size of the central plateau decreases as the core approaches the protostar formation.^9^ Prestellar cores are also characterized by a steep decrease of temperatures toward their center, where the gas reaches temperatures of 6–8 K.^10,11^ As a consequence of the low temperatures and high densities in the center, molecules readily freeze onto dust grains,^12–14^ a process that significantly enhances deuterium fractionation.^3,15^ H_2_D^+^ and the other deuterated isotopologues of H_3_^+^ are the primary sources of deuteration in prestellar cores. They form via the exothermic reaction

$$H_{3}^{+}+\mathrm{HD}\to H_{2}D^{+}+H_{2}$$ 1

that strongly favors H_2_D^+^ production at temperatures below 30 K. Furthermore, the abundance of H_2_D^+^ is also influenced by the *ortho*-to-*para* ratio of H_2_, as the reverse reaction becomes endothermic when H_2_ is predominantly in the *para* form.^16^ H_2_D^+^ is further deuterated by successive reactions with HD, leading to an enhancement of D_2_H^+^ and D_3_^+^.^17^ The deuterated isotopologues of H_3_^+^ are the key players in gas-phase deuteration, while deuterium atoms, formed from the reactive dissociation of deuterated H_3_^+^ isotopologues with electrons, drive the deuteration on the icy surface of dust grains. Overall, very high levels of deuteration have been observed in prestellar cores, where even multiply deuterated molecules are routinely observed, e.g., *c*-C_3_D_2_, D_2_CO, and CHD_2_OH.^18–20^

Complex organic molecules (COMs) are defined as organic molecules with more than five atoms (e.g., ref 21). COMs have been observed in a wide variety of astrophysical environments. In low-mass star-forming regions, they are particularly abundant around protostars, in regions called hot corinos where forming stars heat the surrounding material above the sublimation temperature (100 K) of the water ice mantles on dust grains.^22^ In the past decade, many observations of COMs toward starless and prestellar cores demonstrated that they efficiently form also in very cold environments.^23–28^ According to astrochemical models, COMs in prestellar core centers are mainly present in solid-phase within the thick icy mantles of dust grains (e.g., ref 29), while observable levels of COMs are found in the outskirts of prestellar cores (e.g., refs 26,27 ). Lin et al.^20^ recently reported on the first detection of doubly deuterated methanol (the simplest COM) toward prestellar cores and derived a D/H ratio consistent with measurements in more evolved Class 0/I objects and comet 67P/Churyumov-Gerasimenko,^30^ suggesting a chemical inheritance from the prestellar stage. There is observational evidence suggesting that the chemical budget present in the prestellar phase does not undergo a full reset during protostar formation.^31^ Consequently, prestellar cores act as chemical reservoirs, supplying crucial building blocks for stars and planets. To follow the evolution of prestellar material from one evolutionary stage to the next in the star-formation process, isotopic fractionation proves to be an exceptionally powerful diagnostic tool.^32^ It is, in fact, not possible to reproduce the deuterium fractionation observed in water within the Solar System without taking into account the formation and deuteration of water in the prestellar phase.^33^ Furthermore, recent observations suggest that a fraction of the COMs observed toward protostellar cores are inherited from the prestellar phase.^28,34^ Deuteration maps are a measure of the effectiveness of the deuteration across the core, and they are useful to study both the deuteration as well as the formation of the main species.^35–37^ Furthermore, deuteration maps from multiply deuterated isotopologues (i.e., CHD_2_OH or *c*-C_3_D_2_) are crucial to assess the effects of the spatial distribution of both deuterated and nondeuterated isotopologues on the deuteration peak that results from using the main isotopologue (e.g., CH_2_DOH/CH_3_OH). A clear example is the deuteration of *c*-C_3_H_2_ observed in the prestellar core L1544. While the *c*-C_3_HD/*c*-C_3_H_2_ column density ratio peaks at the east of the dust emission peak, the *c*-C_3_D_2_/*c*-C_3_HD peaks toward the dust peak (see Figure 2 in ref 37). This might suggest that the *c*-C_3_HD/*c*-C_3_H_2_ peak could be a consequence of the steep decrease of the *c*-C_3_H_2_ toward the Northeast in the outer layers of the prestellar cores, rather than a location of enhanced deuteration. In Spezzano et al.,^38^ we showed that the southern part of the prestellar core L1544 is more exposed to the interstellar radiation field (ISRF) and therefore this is where the carbon chain molecules peak. The northeastern part of the core is more shielded, and as a consequence, more carbon will be locked in CO and is not available for the formation of carbon chain molecules. Given that methanol is directly formed from CO on grains,^39^ methanol peaks in the Northeast of L1544. The deuterated isotopologues of *c*-C_3_H_2_, instead, are present only in the inner layers of L1544 and their distribution is not affected by the ISRF.

Observing multiply deuterated molecules is very important to fine-tune our astrochemical models and allow quantitative comparison among the different evolutionary stages in the star- and planet-formation process. With methanol being the simplest O-bearing COM and the starting point of molecular complexity in star-forming regions,^40,41^ understanding its deuteration in prestellar cores will provide crucial constraints on its formation and inheritance in the star-formation process. Although the formation of methanol on dust grains is well-established,^29,39^ the chemical pathways responsible for its deuteration remain unclear, with potential pathways including H-D substitution and hydrogenation of deuterated formaldehyde.^42^ In an effort to identify crucial chemical and physical parameters for the formation and deuteration of methanol in the prestellar phase, Riedel et al.^43,44^ updated a gas-grain chemical code by including various processes such as reactive desorption, diffusion mechanisms for hydrogen and deuterium atoms on the surface of interstellar dust grains, and nondiffusive reaction mechanisms. Such processes are very important to reproduce the observations of COMs in prestellar cores.

In this paper, we explore the deuteration of methanol toward the prototypical presttellar core L1544. This core, located in the Taurus molecular cloud at 170 pc,^45^ is one of the best studied prestellar cores. Its central density is ∼10^6^ cm^–3^ and the central temperature is ∼6 K.^10^ The core exhibits a high degree of CO freeze-out and a high level of deuteration toward its center.^3,12^ It is chemically rich,^25,26^ showing spatial inhomogeneities in the distribution of molecular emission.^46^ For decades, L1544 has been the test bed for studies that have significantly advanced our understanding of the dynamic evolution of dense cores prior to star formation.

The paper is structured as follows: Section 2 presents the observations, and the analysis of the single-dish observations is presented in Section 3. We use state-of-the art chemical models to reproduce the deuteration of methanol in three presttellar cores, and our results are described in Section 4. We discuss the overall results in Section 5 and summarize our conclusions in Section 6.

## 2 Observations

The emission map of the *J*_*K*_a_,*K*_c__ = 2_0,2_-1_0,1_ *e*_0_ transition of CHD_2_OH (*E*_up_ = 6 K) at 83289.63 MHz^47^ toward L1544 was obtained using the IRAM 30 m telescope (Pico Veleta, Spain) in different observing runs between 2022 and 2023 (project codes: 116-21, 043-22, 104-22, PI: S. Spezzano). We performed a 1.4*′* × 1.4*′* on-the-fly (OTF) map centered on the source dust emission peak (α_2000_ = 05^h^04^m^17^s^.21, δ_2000_ = +25°10*′*42″.8). We used position switching with the reference position set at (−180″, 180″) offset with respect to the map center. The EMIR E090 receiver was used with the Fourier transform spectrometer backend (FTS) with a spectral resolution of 50 kHz. The mapping was carried out in good weather conditions (τ_225 GHz_ ∼ 0.3) and a typical system temperature of *T*_sys_ ∼ 90–150 K. The data processing was done using the GILDAS software.^48^ The emission map has a beam size of 30″ and was gridded to a pixel size of 6″ with the CLASS software in the GILDAS package, which corresponds to ∼1/5 of the beam size. The intensity scale was converted into main beam temperature *T*_MB_ assuming forward efficiency *F*_eff_ = 0.95 and *B*_eff_ = 0.81. The noise level was homogeneous in our map; therefore, no weighting was applied to the individual spectra before averaging. While the brightest CH_2_DOH transition in the 3 mm band was observed, the line is still too weak to produce an integrated intensity map. The averaged spectra toward five different regions across L1544 are shown in Figure 1. The single pointing observations toward the dust peak of L1544 shown in Figure 2 are from the IRAM 30 m large program ASAI.^49^ The single pointing observations toward the methanol peak (α_2000_ = 05^h^04^m^18^s^, δ_2000_ = +25°11*′*10″) shown in Figure 2 were obtained with the 30m telescope in 2024 within the framework of project 022-24 (PI: A. Megías).

**Figure 1:**
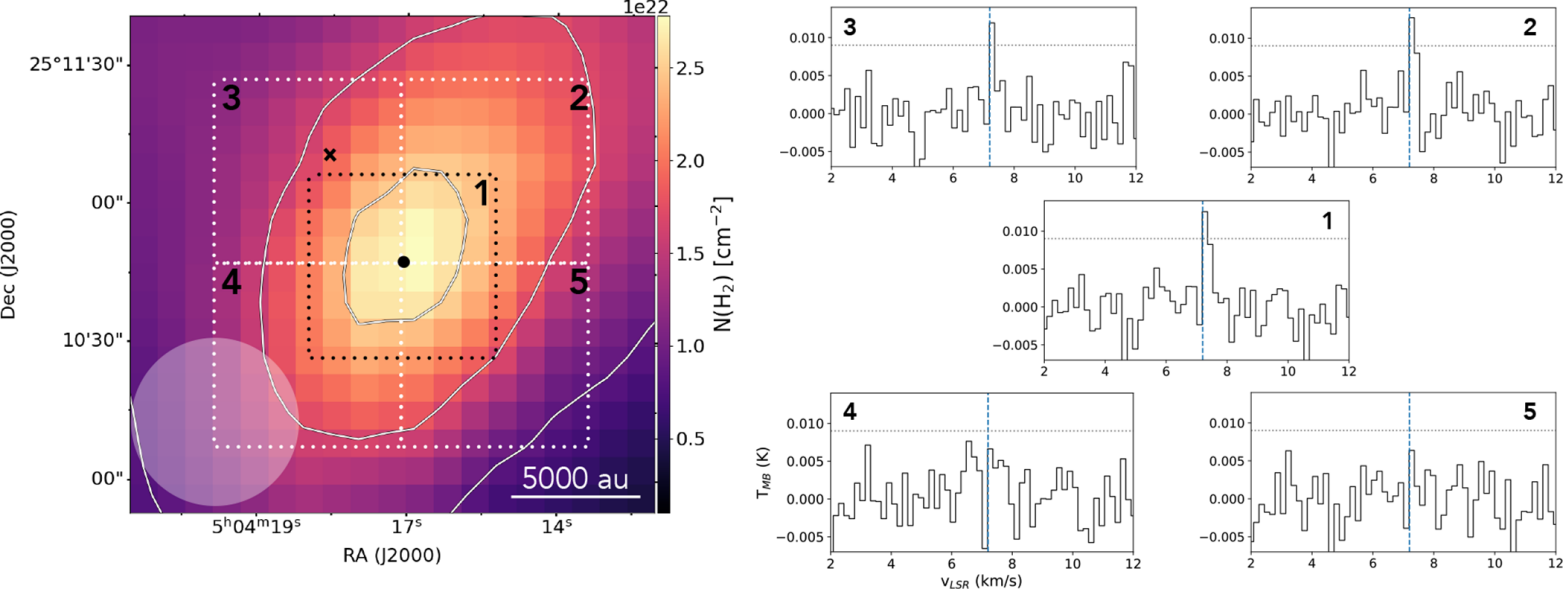
Left panel: the five different areas where the CHD_2_OH spectra have been extracted from the OTF map observed with the IRAM 30 m telescope are shown as dotted squares on the H_2_ column density map of L1544 computed from *Herschel*/SPIRE data at 250, 350, and 500 μm.^38^ The solid white contours are the 30, 60*,* and 90% of the peak intensity of the N­(H_2_) map. The *Herschel*/SPIRE beam is shown at the bottom left of the map. The black saltire shows the position of the methanol peak and the full black circle shows the dust emission peak. Right panel: *J*_*K*_a_,*K*_c__ = 2_0,2_-1_0,1_ *e*_0_ CHD_2_OH spectra extracted from the IRAM 30m OTF map. The vertical dashed lines show the *v*_LSR_ of the source (7.2 km/s), and the horizontal dotted lines show the 3σ noise level. The number in each spectra refers to the area where the spectrum was extracted from, shown in the left panel of the figure.

**Figure 2:**
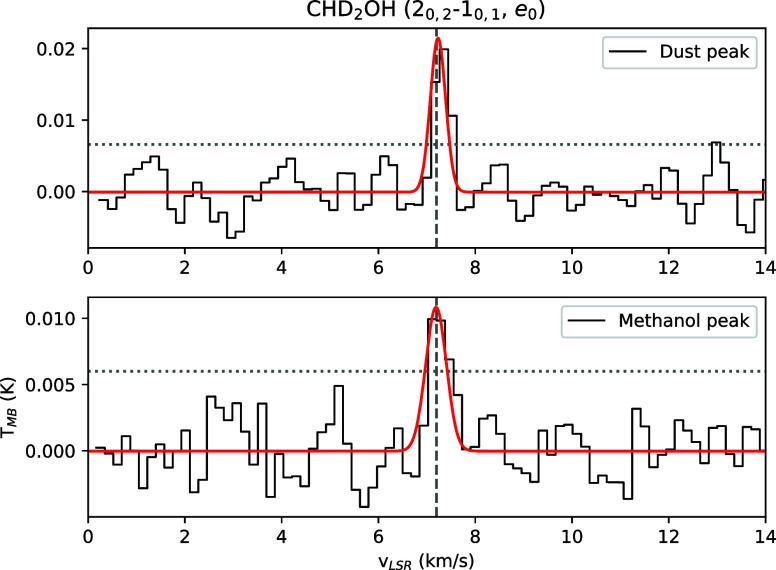
Spectra of CHD_2_OH observed with single pointing observations toward the dust peak (upper panel) and the methanol peak (lower panel) of L1544. The vertical dashed line shows the *v*_LSR_ of the core, 7.2 km/s. The horizontal dotted lines show the 3σ noise level.

## 3 Results

The results of the IRAM 30m project aimed at mapping the 2_0,2_-1_0,1_, *e*_0_ transition of doubly deuterated methanol toward the central 1.4*′* × 1.4*′* region of L1544 are shown in Figure 1. The final rms of the map is ∼5 mK, and the peak intensity of the CHD_2_OH line, toward the dust peak, is ∼15 mK. Given the weakness of the line, we used the OTF data to average the spectra within an area of 40″× 40″ toward five quadrants shown with dotted white lines in Figure 1. All spectra in Figure 1 are plotted between −0.007 and 0.012 K and between 2 and 12 km/s, with the emission lines centered at 7.2 km/s. The color map used as background for the spectra is the N­(H_2_) map of L1544 computed from *Herschel*/SPIRE data.^38^ The spectra in Figure 1 show that the CHD_2_OH line is brightest in the central and the northwest part of the core, where it is detected with S/*N* > 4 over two channels. The line is barely detected in the Northeast (S/N ∼ 4) over only one channel and not detected in the southern part of the core. The result is in agreement with the singly deuterated methanol maps shown in the central and right panels of Figure 2 in Chacón-Tanarro et al.^36^

Although the results shown in Figure 1 allow us to understand the distribution of doubly deuterated methanol in L1544 and qualitatively compare with OTF maps of CH_3_OH, CH_2_DOH, and other deuterated isotopologues observed in L1544,^35–37^ the poor signal-to-noise ratio of the spectra would make a quantitative comparison rather inconclusive. To overcome this limitation, we use deep observations toward the dust and methanol peaks of L1544, where the rms is 2.2 and 2.0 mK, respectively. The single pointing observations are shown in Figure 2. The results of the Gaussian fit toward the dust and methanol peak of L1544 are reported in Table 1. The column densities of CHD_2_OH reported in Table 1 have been computed from the spectra shown in Figure 2 using the formula reported in Mangum and Shirley,^51^ assuming optically thin emission and that the source fills the beam:

$$N_{\mathrm{tot}}=\frac{8\pi \nu^{3}Q_{\mathrm{rot}}(T_{\mathrm{ex}})W}{c^{3}A_{\mathrm{ul}}g_{u}}\frac{e^{E_{u}/kT}}{J(T_{\mathrm{ex}})-J(T_{\mathrm{bg}})}$$ 2

where $J\left(T\right)=\frac{h\nu }{k}{\left(e^{h\nu /kT}-1\right)}^{-1}$ is the Rayleigh-Jeans equivalent temperature in Kelvin, *k* is the Boltzmann constant, ν is the frequency of the line, *h* is the Planck constant, *c* is the speed of light, *A*_ul_ is the Einstein coefficient of the transition, *W* is the integrated intensity, *g*_u_ is the degeneracy of the upper state, *E*_u_ is the energy of the upper state, *Q*_rot_ is the partition function of the molecule at the given temperature *T*_ex_, and *T*_bg_ is the background (2.7 K). We calculated the partition function *Q*(*T*_ex_) at 5, 6.5, and 8 K using the CHD_2_OH catalog from the CDMS,^52^ recently updated based on Drozdovskaya et al.^50^ The resulting partition functions are reported in Table S1. The column densities of doubly deuterated methanol reported in Table 2 were calculated considering variations of *T*_ex_ within 5–8 K and assuming a calibration error of 20% to derive the uncertainties, as done in Lin et al.^20^ Table 2 also reports on the deuteration ratios of methanol at the dust and methanol peaks of L1544, as well as the column densities of the main and singly deuterated isotopologues of methanol reported in Lin et al.^53^ and Chacón-Tanarro et al.,^36^ for completeness. The column densities of the main isotopologue reported in Table 5 of Lin et al.^53^ have been derived with RADEX non-LTE modeling using a total of ten different lines (of which four were observed as upper limits) at 3 and 2 mm and they agree within a factor of 2 with previous values reported in Vastel et al.,^25^ Bizzocchi et al.,^54^ Punanova et al.,^55^ and Chacón-Tanarro et al.^36^

**Table 1:** Parameters of the Observed CHD_2_OH Lines in the Dust and Methanol Peaks of L1544^a^

	*W* mK km s^–1^	*v*_LSR_ km s^–1^	fwhm km s^–1^	rms mK	*N*_TOT_ (*T*_ex_ = 5 K) cm^–2^	*N*_TOT_ (*T*_ex_ = 6.5 K) cm^–2^	*N*_TOT_ (*T*_ex_ = 8 K) cm^–2^
dust peak	8.4(9)	7.19(2)	0.37(4)	3	6.7(7) × 10^11^	7.0(8) × 10^11^	8.0(9) × 10^11^
methanol peak	6.2(9)	7.18(5)	0.47(9)	3	5.0(7) × 10^11^	5.2(8) × 10^11^	5.9(9) × 10^11^

a Note: The laboratory spectroscopy reference for CHD_2_OH is Drozdovskaya et al.^50^ The integrated intensities are reported in units of *T*_MB_. Numbers in parentheses denote 1σ uncertainties in units of the last quoted digit.

**Table 2:** Column Densities and Column Density Ratios at the Dust Peak and Methanol Peak of L1544

	dust peak	methanol peak
*N*(CH_3_OH)^a^	1.30(5) × 10^13^ cm^–2^	1.60(3) × 10^13^ cm^–2^
*N*(CH_2_DOH)^b^	2.8(7) × 10^12^ cm^–2^	3.3(8) × 10^12^ cm^–2^
*N*(CHD_2_OH)^c^	7.2(1.4) × 10^11^ cm^–2^	5.4(1.5) × 10^11^ cm^–2^
*N*(CH_2_DOH)/*N*(CH_3_OH)	22(6)%	21(5)%
*N*(CHD_2_OH)/*N*(CH_3_OH)	6(1)%	3(1)%
*N*(CHD_2_OH)/*N*(CH_2_DOH)	26(8)%	16(6)%

a From ref 53.

b From ref 36.

c This work. Numbers in parentheses denote 1σ uncertainties in units of the last quoted digit.

## 4 Chemical Models

To compare if our current theoretical understanding of deuterium chemistry can match the observed deuteration trends for methanol, we have tested several models originally developed to reproduce the CH_2_DOH/CH_3_OH ratio.^43,44^

The chemical evolution of molecular abundances is computed with the gas-grain astrochemical code *pyRate*.^56,57^ The chemical network for the gas-phase is based on the 2014 public release of the Kinetic Database for Astrochemistry.^58^ A recent update to the latest data release (kida.uva.2024, ref 59) was tested on a 0D model using a nondeuterated chemical network and showed no significant deviations for methanol, and hence for simplicity, we decided to proceed with the existing deuterated networks.^43,44^ The grain surface network is based on the one presented in Semenov et al.^60^ Reactions were cloned to include deuterated counterparts for species of up to seven atoms and spin-state counterparts for selected species. Uncertainties arise when reactions are cloned to describe the evolution of the deuterated species. The methanol formation and deuteration scheme follows the experimentally verified proposal by Hidaka et al.^42^ Generally, experimental data is used when it is available; for details on the network and deuteration schemes, we refer to Riedel et al.^44^ and references therein. The models assume a three-phase grain model, including a gas-phase, a chemically active surface phase, and an inert mantle-phase. Desorption of methanol from the surface of the dust grain occurs predominantly through nonthermal desorption mechanisms in the extremely cold conditions of prestellar cores. Usually, reactive desorption is presumed to be the dominant one.^29,43^ All models presented in this work apply a constant reactive desorption efficiency of 1%.^61^ The formation enthalpies and binding energies used in the model are reported in Table A.1 of Riedel et al.^43^ We note that a few binding energies listed in Table A.1 of Riedel et al.^43^ have been recently revised in theoretical and experimental studies (e.g., ref 62). However, in cold environments such as L1544, binding energies exceeding ∼2000 K are too high for thermal or CR-induced desorption to have a substantial effect on gas-phase abundances. After a recent update,^44^ pyRate includes several nondiffusive reaction mechanisms. However, their impact on the chemistry of methanol, which is mainly formed and deuterated by addition and abstraction reactions of highly mobile H and D atoms, was found to be only minor in Riedel et al.,^44^ where a factor of nondiffusive/diffusive of 1.07 (dust peak) and 0.95 (methanol peak) is derived at the best fit-time (*t* = 3 × 10^5^ yr). The models presented in this work therefore include solely the more conservative diffusive chemistry. Nonetheless, we note that Jiménez-Serra et al.^63^ found that the formation of CO, CO_2_, and CH_3_OH is tightly linked, so that nondiffusive chemistry may lead to some different results. Surface reactions proceed through the Langmuir–Hinshelwood mechanism, relying on thermal diffusion. Riedel et al.^44^ tested over 30 different models while investigating the formation and deuteration of methanol in cold dense cores, like L1544. Here, we compare the result of the best four models (D2, D3, D4, and D5) against our observations in L1544. To facilitate the comparison, we kept the same nomenclature as in Riedel et al.^44^ The characteristics of the models used in this article are listed in Table S2. Models D2, D3, and D4 adopt only H-addition reactions, while model D5 also includes H-abstraction reactions. Model D2 additionally allows for the diffusion of hydrogen and deuterium atoms by quantum tunneling through a rectangular barrier of 1 Å width. The diffusion-to-binding energy *E*_d_/*E*_b_ is set to 0.55; with the exception of model D3, where it is set to 0.2, the lowest value debated in the literature.^64^ Reactions with an activation-energy barrier play an important role in the hydrogenation (and deuteration) of methanol. Hence, the approach used to derive their reaction probabilities has a significant effect on the formation of methanol and its deuterated isotopologues. Here, we test two approaches widely used in the literature. Models D2 and D3 apply the single collision approach,^65^ which assumes that the reactant has only one attempt to either thermally hop over the barrier or tunnel through it. Models D4 and D5 apply the reaction-diffusion competition approach,^66^ which considers that the reaction partners are confined in the same binding site until one of them diffuses away again and can therefore undergo multiple attempts to react with each other. The chemical models were run using the physical structure of L1544,^67^ shown in Figure S2. All models use the initial chemical abundances reported in Table 1 of Riedel et al.,^44^ considering a spherical dust grain with a radius of 0.1 μm and a surface density of binding sites of 1.5 × 10^15^ cm^–2^. This work uses the canonical value for ζ_2_ (1.3 × 10^–17^ s^–1^). We note that a recent re-evaluation has been presented in Redaelli et al.,^68^ and the revised value is consistent within the uncertainty of the method with the canonical value of 1.3 × 10^–17^ s^–1^. The visual extinction in the models is calculated as *A*_V_ = 10^–21^ *N*(H_2_); a floor value of 1 mag for L1544 and L694-2 and 3 mag for HMM1 is added to account for the more extended envelope. The external values used for the three cores are 1 mag for L1544 and L694-2 and 3 mag for HMM1. The resulting abundances were converted to column densities, including beam convolution with a beam size corresponding to the observations. The results of the models for L1544 and the comparison with the observations are shown in Figure 3.

**Figure 3:**
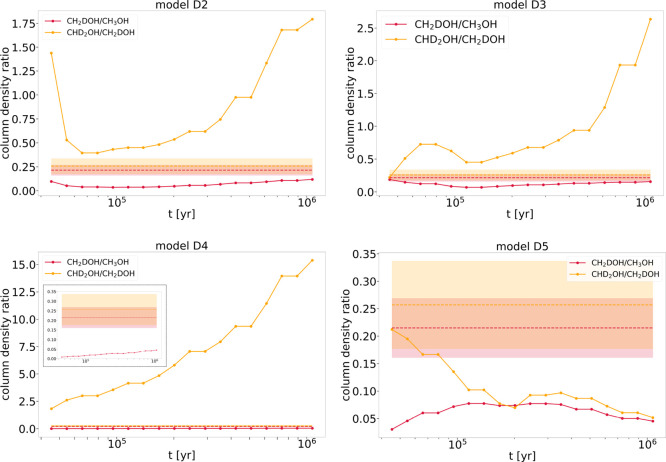
Column density ratios for the deuteration of methanol in L1544 computed with four of the models presented in Riedel et al.^44^ The horizontal dashed lines show the result from the observations toward the dust peak of L1544, and the shaded region indicates the error bars of the observed ratios. Models D2 and D3 apply the single collision model proposed by Hasegawa et al.^65^ with either tunnel diffusion (D2) or fast diffusion (D3). Models D4 and D5 apply the reaction-diffusion competition model proposed by Chang et al.^66^ Additionally, D5 allows for H-abstraction reactions. For model D4, a zoom-in for low values of column density ratios was added within the plot.

## 5 Discussion

The spectra on the L1544 map in Figure 1 show that the line of CHD_2_OH is not detected toward the southern part of the core, and detected at a 2σ level (in integrated intensity) toward the northeast part of the core. The distribution of methanol in L1544 is characterized by a sharp decrease toward the South because of a more efficient illumination from the interstellar radiation field.^38^ It is therefore not surprising that we do not observe CHD_2_OH in the southern part of the core. On the contrary, it might be surprising that the line is very weak toward the northeast part of the core, given that the methanol peak is located toward the northeast with respect to the dust peak of L1544.^54^ It is important to note, however, that the quadrants that we used to average the spectra shown in Figure 1 are large, and the position of the methanol peak is covered by both the central and the upper-left quadrant. Overall, the spectra in Figure 1 show that our target line for CHD_2_OH is observed in a rather small portion of the map around and slightly toward the north of the dust peak.

When comparing the deuteration of methanol toward the dust and methanol peaks in L1544 using the deep observations shown in Figure 2, we do not see significant differences within error bars. The deuterium fractions measured toward the dust peak, however, tend to be larger than the ones measured toward the methanol peak, as reported in Table 2. The deuteration maps of N_2_H^+^, HCO^+^, and *c*-C_3_H_2_ in L1544 also peak toward the center of the core (e.g., refs 35,37 ) where the deuteration is more efficient because of the local increase in the abundance of H_2_D^+^, D_2_H^+^, and D_3_^+^, as well as the catastrophic freeze-out of CO.^12^ The level of deuteration reached by each molecule varies, and it is likely influenced both by the molecule’s distribution within the different layers of the core,^35,38^ as well as by the relevant deuteration processes for each molecule. N_2_H^+^ has larger levels of deuteration (26%) than *c*-C_3_H_2_ (17%) and HCO^+^ (3.5%) because it traces best the denser gas in the center of L1544, where the deuteration is more efficient. The level of deuteration measured in methanol is similar to N_2_H^+^ even if methanol traces an outer shell of L1544,^25,38,54^ as *c*-C_3_H_2_ does. This is indicative of a much more efficient deuteration process taking place in the interstellar ices, where methanol and deuterated methanol are formed, in comparison with the deuteration happening in the gas-phase (e.g., for *c*-C_3_H_2_). The deuteration ratio R_D_ = *N*(XHD)/*N*(XH_2_) is ∼15% for *c*-C_3_H_2_ and ∼20% for methanol, while the R_D_2__ = *N*(XD_2_)/*N*(XHD) is ∼1% for *c*-C_3_H_2_ and ∼25% for methanol, indicating that the second deuteration of methanol, to form CHD_2_OH, is also more efficient than the second deuteration of *c*-C_3_H_2_, to form *c*-C_3_D_2_. Particularly puzzling is the deuteration of H_2_CO and H_2_CS, whose large R_D_2__ (∼100%) toward the dust peak of L1544. While, unlike methanol, H_2_CO and H_2_CS can also be formed and deuterated in the gas-phase (e.g., ref 75), the large R_D_2__ observed in L1544 cannot be reproduced with chemical models that consider the deuteration on the surface as well as in the gas-phase.^36,76^

To understand the different deuterium fractions observed in L1544, we use the best models among the ones developed and tested by Riedel et al. for L1544, as described in Section 4, and compared the results against the observed trends. The results shown in Figure 3 clearly indicate that model D5 is the only one that does not predict very large R_D_2__ ratios that would strongly disagree with our observations. Additionally, model D5 predicts relatively similar values for R_D_2__ and R_D_, which is in agreement with our observations. This is a very interesting result because model D5 is the only one that includes H-abstraction reactions. H-abstraction reactions have been studied in the laboratory^42,77,78^ and the experimental results showed their importance in the reaction scheme for methanol formation.

Lin et al.^20^ reported on the first detection of doubly deuterated methanol toward prestellar cores and observed deuterium fractions toward L694-2 and HMM-1 that are different than what we observe in L1544. The R_D_ is 3% in L694-2 and 6% in HMM-1, lower than what we observe for L1544 (20%). On the other hand, R_D_2__ is 50% in L694–2 and 80% in HMM-1, larger than what we observe in L1544 (25%). Figure S1 shows the results of the chemical modeling using model D5 on L694-2 and HMM-1, with the physical structure of the core being the only difference when applying model D5 to the different cores in our sample. It is very interesting to note the effect that the different physical structures of the three cores (L1544, L694-2, and HMM1), shown in Figure S2, have on the predicted ratios. Figure S3 shows the results of the D5 models for the three cores to facilitate the comparison among them. Additionally, it is worth noticing that the observed R_D_2__ and R_D_ ratios in L694–2 and HMM1 can also be well reproduced within a factor of 2.

In Figure 4, we have included our results on L1544 in the plots shown in Figure 2 of Lin et al.^20^ The summary plots in Figure 4 show the values of R_D,_ and R_D_2__ reported in the literature for starless and prestellar cores, protostars, and comets. As already discussed in Lin et al., there is strong observational evidence that the deuteration of methanol is enhanced in dynamically evolved cores and that the presttellar methanol is efficiently inherited in the protostellar phase. R_D_2__ shows the least variations across the sources in Figure 4 because singly and doubly deuterated methanol are more likely to trace the same gas, while the normal isotopologue is also present in regions of the cores where deuteration is not efficient. A non-LTE analysis for the excitation of CH_3_OH was considered for the starless and prestellar cores in Figure 4, while for the other objects in Figure 4, the analysis for CH_3_OH was done under the assumption of LTE. The differences in R_*D*_ that arise from using the LTE vs non-LTE analysis can be significant. In the case of L1544, for example, R_D_ is 7(2)% assuming LTE,^36^ while we derive here a value of 22(6)% using the column density of CH_3_OH computed with a non-LTE analysis in Lin et al.^53^ As a consequence, the values shown in Figure 4 for the protostars and the comet may differ by a factor of 3.

**Figure 4:**
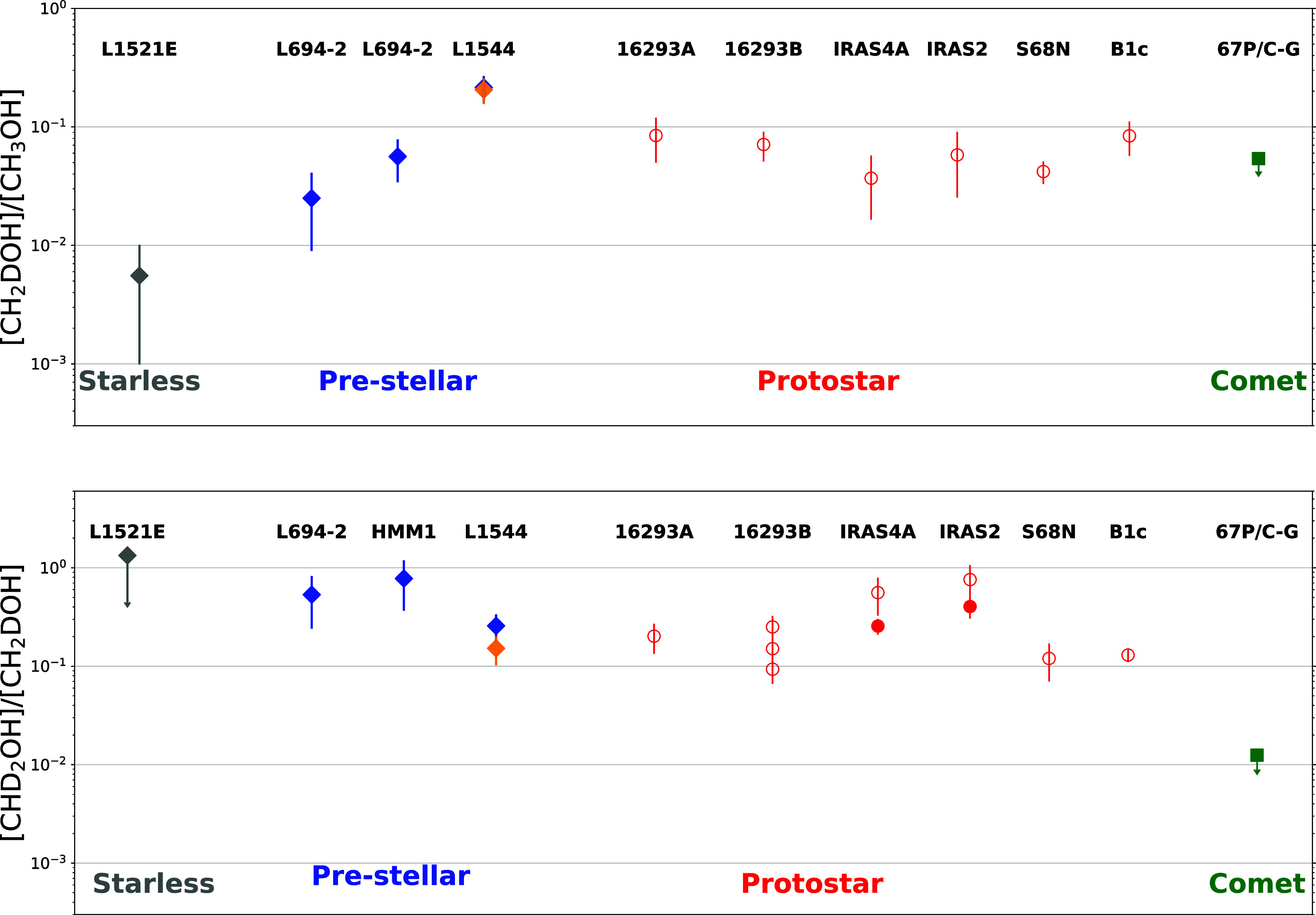
Column density ratios of [CH_2_DOH]/[CH_3_OH] (upper panel) and [CHD_2_OH]/[CH_2_DOH] (lower panel) as a function of source types, rearranged from Lin et al.^20^ with L1544 values in the dust (blue) and methanol (orange) peaks (from this work). Filled markers indicate single-dish observations, and open markers indicate interferometric observations. The plotted values for L1544 take into consideration T_ex_ variations from 5 to 8 K and use the CH_3_OH column density derived in Lin et al.^69^ with non-LTE models. The references for S68N and B1c are from van Gelder et al.;^70^ for IRAS4A and IRAS2 from Taquet et al. and Parise et al.;^71,72^ for IRAS16293A and IRAS16293B from Manigand et al.,^73^ Jo̷rgensen et al.,^74^ and Drozdovskaya et al.; and^50^ for comet 67P/C-G from Drozdovskaya et al.^30^

## 6 Conclusions

Isotopic fractionation, and in particular, deuteration, is an excellent tool to understand the formation and inheritance of molecules in star-forming regions. Toward the prestellar core L1544, methanol exhibits levels of deuteration as large as N_2_H^+^, highlighting its very efficient deuteration on the icy surface of dust grains.

By comparing our observational results with state-of-the-art chemical models, we can gauge the importance of H-abstraction reactions in the formation and deuteration of methanol on the surface of dust grains. Additionally, we have compared the observations of three prestellar cores and assessed the large effect that their physical structures have on the deuteration of methanol.

Collisional rate coefficients for deuterated methanol will be necessary to assess non-LTE effects and the consequent effects on the column density that we routinely derive in star-forming regions.

Methanol represents the beginning of molecular complexity in star-forming regions; thus, a complete understanding of its formation and deuteration is a necessary step to understand the development of further chemical complexity. In this regard, understanding the processes responsible for the very high R_D_2__ measured in H_2_CO, an intermediate in the formation of methanol on the surface of dust grains, is of paramount importance.

## Supplementary Material



## Notes

Spectroscopy and catalogs of deuterated methanol and dependence of deuteration on physical conditions (PDF)
