# Supplementary material for: High Deuteration of Methanol in L1544
Source: ACS Earth Space Chem. 2025 Dec 22;10(1):57–65. doi: 10.1021/acsearthspacechem.5c00187 (PMC12814770; doi:10.1021/acsearthspacechem.5c00187)
Supplement: Supplementary file 1 [file sp5c00187_si_001.pdf]

Supporting Information

High deuteration of methanol in L1544†

Silvia Spezzano[1*], Wiebke Riedel[1], Paola Caselli[1], Olli Sipilä[1], Yuxin Lin[1], Hayley A. Bunn[1], Elena Redaelli[2], Laurent H. Coudert[3], Andrés Megías[4], Izaskun Jimenez-Serra[4]

[1]*Max-Planck-Institut für Extraterrestrische Physik, Giessenbachstrasse 1, 85748 Garching, Germany;* [2]*European Southern Observatory, Karl-Schwarzschild-Strasse 2, 85748 Garching, Germany;* [3]*Institut des Sciences Moléculaires d'Orsay (ISMO), CNRS, Université Paris-Saclay, F-91405 Orsay, France;* [4]*Centro de Astrobiología (CAB), CSIC-INTA, Carretera de Ajalvir, km 4, 28805, Torrejón de Ardoz, Spain*

E-mail: spezzano@mpe.mpg.de

On the spectroscopy and catalogs of deuterated methanol

The internal rotation of the asymmetric methyl group in the isotopologues of methanol with deuterium in the methyl group leads to complex spectral patterns that require complex analysis. As a consequence, particular care needs to be taken when using data from online catalogs as approximate methods used to produce the catalog might have effects on the interpretation of astronomical data. In the JPL catalog, the rest frequencies of CH_2DOH transitions that are energetically favorable to observe in cold sources like starless and pre-stellar cores have errors in the order of ∼100 kHz with respect to the measured frequencies reported in the supplementary material of Coudert

†Based on observations carried out with the IRAM 30 m telescope. IRAM is supported by INSU/CNRS (France), MPG (Germany), and IGN (Spain).

et al. (2014).[1] Such small deviations are significant in cold sources because of the characteristic small line-widths and might induce significant error in the velocity of the line or even misidentification. We therefore suggest to refer to the rest frequencies reported in the spectroscopy papers and compare them to the catalogs, especially in cases of doubts on the interpretation of the astronomical data. The complexity of the rotational ladder also translates into potential errors when extrapolating the values of their partition functions at temperatures not listed in the online catalogs. We therefore list in Table S1 the partition functions Q(T) for CH_2DOH and CHD_2OH in a large range of temperatures, including temperatures relevant for cold sources like starless and pre-stellar cores. The values reported in Table S1 have been computed using all torsional levels up to 1700 cm^{-1} for CH_2DOH and 2000 cm^{-1} for CHD_2OH. We note that the partition function reported in Table S1 for CH_2DOH is very close to the values currently listed in the JPL catalog, despite the catalog only listing transitions from the ground torsional state based on Pearson et al. (2012).[2] It is plausible that a correction factor has been used to correct the partition functions listed in the JPL catalog (Drouin, priv. comm.). This warrants a re-evaluation of the column densities derived with the JPL partition function that used a correction factor (e.g. Jørgensen et al. 2018[3]).

Table S1: Partition function, Q(T), of CH_2DOH and CHD_2OH

T(K)	CH_2DOH	CHD_2OH
300	16753.496	19331.489
225	9506.242	11256.666
150	4385.771	5255.582
75.0	1294.257	1563.391
37.5	398.999	490.483
18.75	114.589	145.295
9.375	30.388	39.851
8.0	22.447	29.521
6.5	15.210	20.009
5.0	9.603	12.412

Table S2: Overview of the four models from Riedel et al. (2025)[4] used in this work.

Model	RD[a] efficiency	H_2 removal[b]	other modifications
D2⋆	1%	scaled E_b	tunnel diffusion
D3⋆	1%	scaled E_b	fast diffusion
D4†	1%	scaled E_b	
D5†	1%	scaled E_b	H-abstraction reac.

Note: ⋆ Models that apply the single collision model proposed by Hasegawa et al. (1992)[5] to derive the reaction probabilities. † Models that apply the reaction-diffusion competition model proposed by Chang et al. (2007)[6] to derive the reaction probabilites. [a] Efficiency of the reactive desorption. [b] To avoid unphysical build-up of H_2 (and its deuterated isotopologues) on the surface, their binding energies have been scaled by a factor of 0.1.[4]

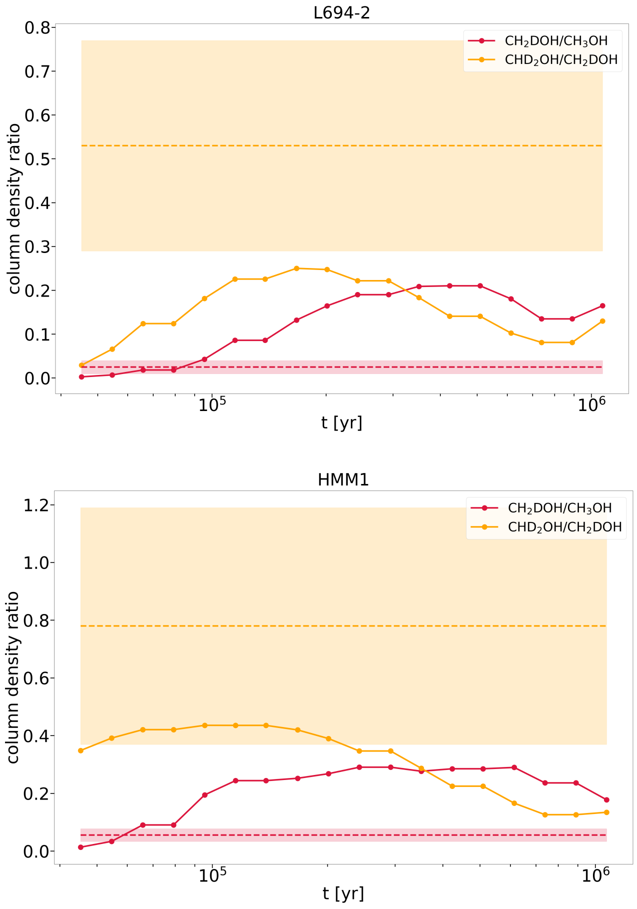

Figure S1: Results from the best model (D5) from Riedel et al. (2025)[4] for the pre-stellar cores L694-2 and HMM-1. The horizontal dashed lines show the result from the observations and the shaded region indicates the error bars of the observed ratio.[7]

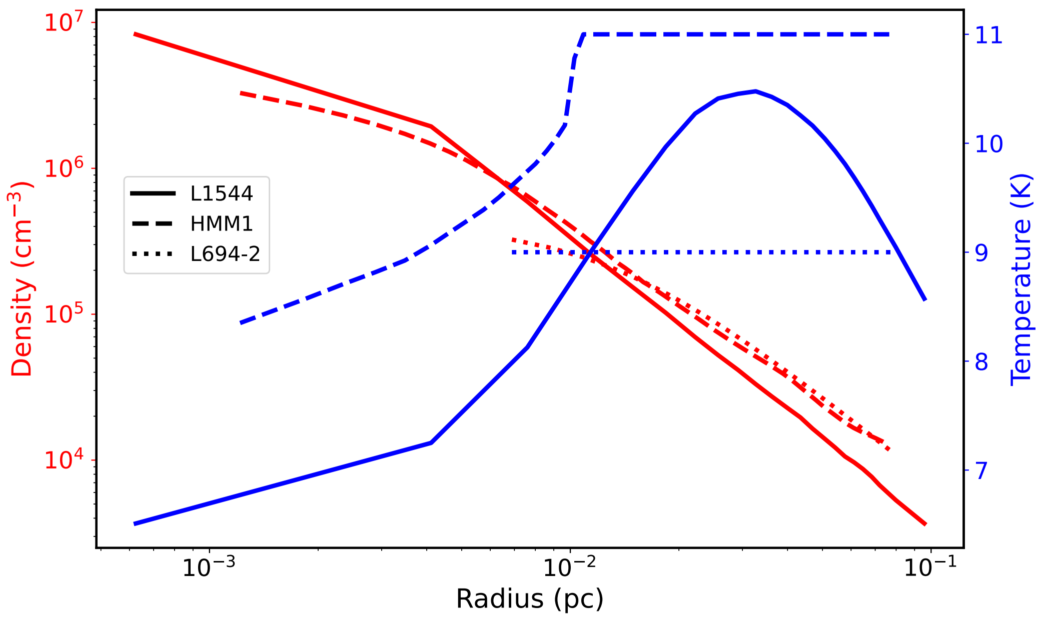

Figure S2: Physical structures of L1544,[8] HMM1,[9,10] and L694-2[11] used for the chemical models.

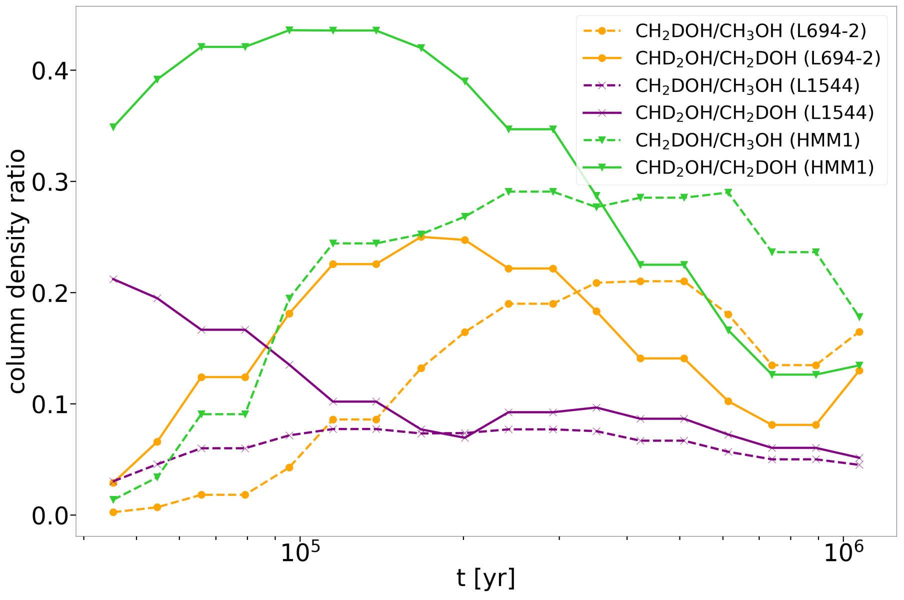

Figure S3: R_D and R_{D_2} for methanol predicted by the model D5 from Riedel et al. (2025) using the physical structures of L1544,[8] HMM1,[9,10] and L694-2.[11]

Dependence of deuteration on physical conditions

The dependence of the modelled R_D and R_{D_2} on the physical properties, mainly the H_2 density and temperature, is a complicated multidimensional problem and a detailed exploration is beyond the scope of this paper.

The fractionation of the deuterium in the gas phase is most efficient in the cold and dense center of the pre-stellar core. H and D atoms are directly adsorbed onto the surface of dust grains from the gas phase, where the atomic D/H ratio is enhanced. However, an increased atomic D/H ratio on the grain by itself does not guarantee, that the deuterium atoms are able to meet their reaction partners successfully. This also depends on the efficiency of the diffusion process, that is dictated by the employed mode of diffusion and the grain temperature. In general, higher grain temperatures allow for a faster diffusion process and a higher reaction rate of potential reaction partners.

To shed some light into the density and temperature dependence, we have run a grid of static 0D simulations (see Figure S4 and S5). The H_2 densities range from 3×10^6 cm^{-3} to 9×10^6 cm^{-3}. The gas and grain temperatures are set to the same value and range from 6.0 K to 9.0 K. The selected range is an appropriate parameter range for the dust peak of the three pre-stellar cores (L1544, HMM1 and L694-2) investigated. This is where the highest levels of deuteration are expected to occur. All other parameters remain constant between individual runs.

We conclude that both H_2 density and temperature affect the R_D and R_{D_2}. The highest R_D and R_{D_2} ratios are determined for high densities $n(H_2) = 9 \times 10^6$ cm^{-3}, where fast gas phase reactions promote an efficient fractionation process and short freeze-out timescales quickly deliver the atomic R_D to the grain's surface, and high $T_{grain} = 9$ K speed up the diffusion process. We also note that partially similar R_D and R_{D_2} can be obtained by multiple parameter pairings.

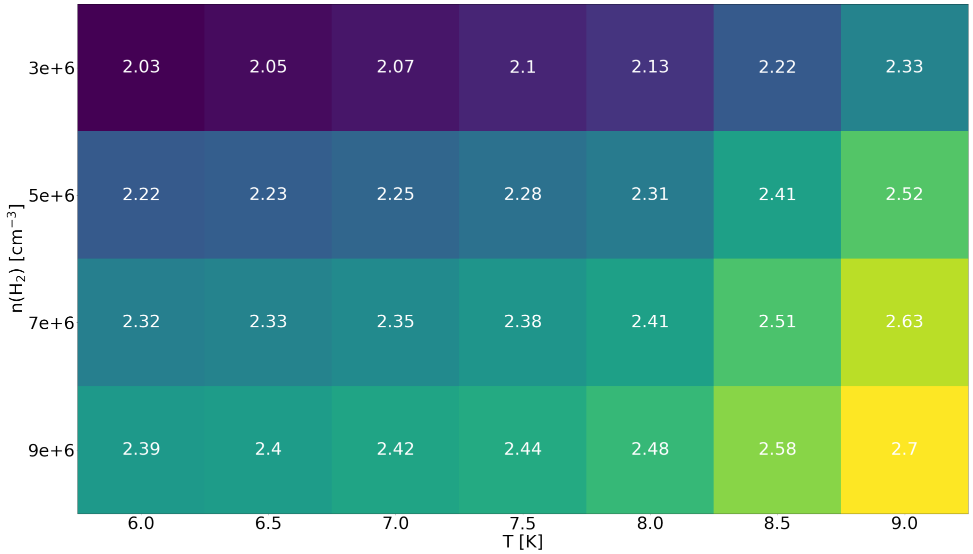

Figure S4: Heatmap of the abundance ratio of CH_2DOH over CH_3OH for a parameter grid with $n(H_2)$ between 3×10^6 cm^{-3} to 9×10^6 cm^{-3} and T_{gas} and T_{grain} between 6.0 K and 9.0 K.

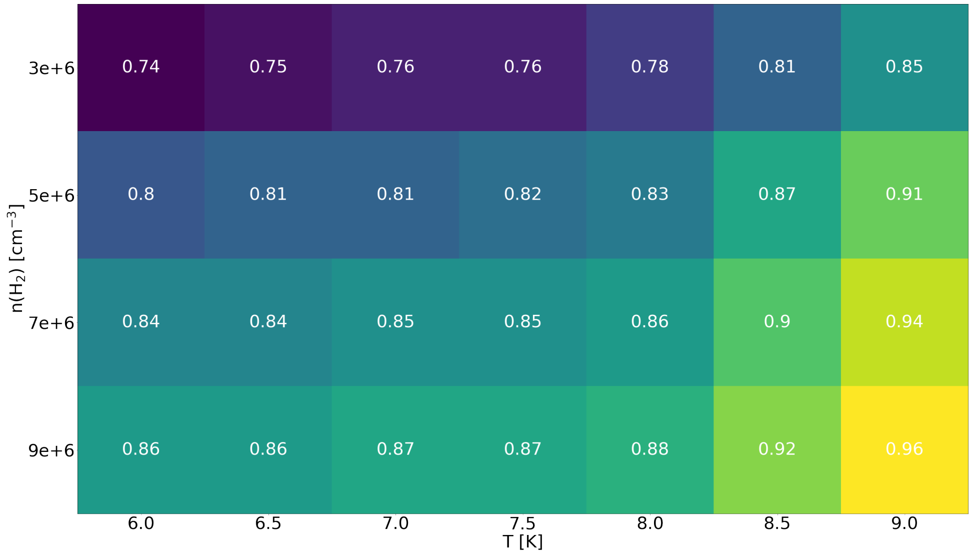

Figure S5: Heatmap of the abundance ratio of CHD_2OH over CH_2DOH for a parameter grid with $n(H_2)$ between 3×10^6 cm^{-3} to 9×10^6 cm^{-3} and T_{gas} and T_{grain} between 6.0 K and 9.0 K.

References

(1) Coudert, L. H.; Zemouli, M.; Motiyenko, R. A.; Margulès, L.; Klee, S. Analysis of the mi-

crowave, terahertz, and far infrared spectra of monodeuterated methanol CH_2DOH up to J = 26, K = 11, and o_1. *JCP* **2014**, *140*, 064307.

(2) Pearson, J. C.; Yu, S.; Drouin, B. J. The ground state torsion rotation spectrum of CH_2DOH. *J. Mol. Spectrosc.* **2012**, *280*, 119–133.

(3) Jørgensen, J. K.; Müller, H. S. P.; Calcutt, H.; Coutens, A.; Drozdovskaya, M. N.; Öberg, K. I.; Persson, M. V.; Taquet, V.; van Dishoeck, E. F.; Wampfler, S. F. The ALMA-PILS survey: isotopic composition of oxygen-containing complex organic molecules toward IRAS 16293-2422B. *A&A* **2018**, *620*, A170.

(4) Riedel, W.; Sipilä, O.; Redaelli, E.; Jin, M.; Vasyunin, A. I.; Garrod, R. T.; Caselli, P. Forming deuterated methanol in pre-stellar core conditions. *A&A* **2025**, *701*, A291.

(5) Hasegawa, T. I.; Herbst, E.; Leung, C. M. Models of Gas-Grain Chemistry in Dense Interstellar Clouds with Complex Organic Molecules. *ApJSS* **1992**, *82*, 167.

(6) Chang, Q.; Cuppen, H. M.; Herbst, E. Gas-grain chemistry in cold interstellar cloud cores with a microscopic Monte Carlo approach to surface chemistry. *A&A* **2007**, *469*, 973–983.

(7) Lin, Y.; Spezzano, S.; Caselli, P. First detection of CHD_2OH towards pre-stellar cores. *A&A* **2023**, *669*, L6.

(8) Keto, E.; Caselli, P.; Rawlings, J. The dynamics of collapsing cores and star formation. *MNRAS* **2015**, *446*, 3731–3740.

(9) Pineda, J. E. et al. An Interferometric View of H-MM1. I. Direct Observation of NH_3 Depletion. *AJ* **2022**, *163*, 294.

(10) Harju, J.; Vastel, C.; Sipilä, O.; Redaelli, E.; Caselli, P.; Pineda, J. E.; Belloche, A.; Wyrowski, F. A low cosmic-ray ionisation rate in the pre-stellar core Ophiuchus/H-MM1. Mapping of the molecular ions ortho-H_2D^+, N_2H^+, and DCO^+. *A&A* **2024**, *688*, A117.

(11) Lin, Y.; Spezzano, S.; Pineda, J. E.; Harju, J.; Schmiedeke, A.; Jiao, S.; Liu, H. B.; Caselli, P. Initial conditions of star formation at $\lesssim$2000 au: Physical structure and NH_3 depletion of three early-stage cores. *A&A* **2023**, *680*, A43.
